# Case of Acites, Cured by Injection of a Stimulating Fluid into the Peritoneal Cavity

**Published:** 1845-11

**Authors:** 


*Case of Ascites, cured by the Injection of a Stimulating Fluid into the Peritoneal Cavity.*—Mrs. Newman, of Warren county, N. J., aged 40, mother of eight children, had been in declining health for a year or more previous to Dr. Clark's seeing her; a short time before which she was supposed to be again with child. Her debility and emaciation increased, and also a distension of the abdomen, added to which she had a prolapsus of the bladder. Dr. Clark saw her for the first time on the 30th of November, 1843; he found her in this miserable condition, with poor appetite and fever, suffering constant uneasiness while sitting, and pain when on her feet. All ordinary remedial medical agents were used to no purpose, and on the 14th of December, Dr. C. deemed it necessary to perform paracentesis abdominis, and he drew off 5½ gallons. She bore the operation well. Emaciation, however, continued to advance. She now submitted to small bleedings, and the belly filled more slowly. On the 4th of January, 1844, she was again tapped, and gave 3½ gallons; her decline was now more rapid; no appetite, and great emaciation. It now seemed evident to Dr. C. that but one more tapping could be borne—he considered that the debility induced by the operation would so lessen the liability to inflammation, that he felt justified in injecting an astringent infusion, and thus produce some alterative effects upon the secreting surface of the peritoneum. On the 23d of February, she was subjected to the operation the third time; 3 gallons were removed. She was now very prostrate, requiring powerful stimuli. Her physician had prepared an infusion of the dried sliced fruit of the persimmon, [Diospyros Virginiana); with this he charged a ten ounce syringe, to which he attached a large sized catheter. This he introduced several inches into the wound in the abdomen—he allowed it to remain in ten minutes, when the belly was emptied by pressure upon its walls. The doctor continued his personal attendance at the bedside 24 hours. Prostration was extreme; but reaction became established at the end of 24 hours, and in 36 she had some fever, and great tenderness of the abdomen. She could not move nor speak above a whisper during the first 36 hours. Tepid fomentations were applied to the abdomen, and continued until a bandage could be borne.

A profuse bronchorrhœa had now set in, and in an hour a large silk handkerchief would become saturated. This was on the third day after the last tapping—it was checked by inhalations of chlorine—this drove the water from the lungs to the skin. The diaphoresis becoming too profuse, it was stopped principally with lime water, and frictions with pepper and brandy. After four or five days, the discharge from the lungs returned, and a similar medication drove it again to the skin. The same applications were re-applied, and at the same time the inhalations. During the metastases, the water discharged decreased in quantity, and the patient's appetite increased. A gastrorrhœa now occurred; constant nausea, frequent retching, and some vomiting at intervals. An emetic was given, and the morbid tendency seemed overcome. The urinary secretion became fully established, and she recovered, so that by the 10th of June, 1844, all her functions were fully restored, and since that time she has enjoyed perfect health.

I make no comment on the case, and would merely call attention to the recent experiments of M. Velpeau, an account of which was given in a number of the American Journal of last year.

The report of the above case was placed at my disposal by Dr. William C. Clark, an eminent practitioner of twenty years standing, and who had charge of the patient.—*American Journal of Medical Science.*

